# Cancer patients with COVID-19: a retrospective study of 51 patients in the district of Piacenza, Northern Italy

**DOI:** 10.2144/fsoa-2020-0157

**Published:** 2020-11-24

**Authors:** Luigi Cavanna, Chiara Citterio, Ilaria Toscani, Cosimo Franco, Andrea Magnacavallo, Serena Caprioli, Evelina Cattadori, Camilla Di Nunzio, Roberto Pane, Roberta Schiavo, Claudia Biasini, Massimo Ambroggi

**Affiliations:** 1 Oncology & Haematology Department, Oncology Unit, Piacenza General Hospital, Via Taverna 49, 29121 Piacenza, Italy; 2 Respiratory Intensive Care Unit, Piacenza General Hospital, Via Taverna 49, 29121 Piacenza, Italy; 3 Emergency Department, Piacenza General Hospital, Via Taverna 49, 29121 Piacenza, Italy; 4 Administration Unit, Piacenza General Hospital, Via Taverna 49, 29121 Piacenza, Italy; 5 Research & Innovation Unit, Piacenza General Hospital, Via Taverna 49, 29121 Piacenza, Italy; 6 Pharmacy Unit, Piacenza General Hospital, Via Taverna 49, 29121 Piacenza, Italy; 7 Pathology Unit, Piacenza General Hospital, Via Taverna 49, 29121 Piacenza, Italy

**Keywords:** cancer, COVID-19, hydroxychloroquine, infection, mortality, SARS-COV-2

## Abstract

**Background:**

Cancer patients are considered a highly fragile group in the current coronavirus disease 2019 (COVID-19) pandemic.

**Material & methods:**

In this study, patients with COVID-19 and cancer, hospitalized in Piacenza, Italy, from 4 April to 4 May 2020 were included. Risk factors for death were analyzed.

**Results:**

Fifty-one COVID-19 cancer patients were included, of which the median age was 71.02 years (range: 51–86) and 70.59% were male. Cancer types included gastrointestinal (25.49%), genitourinary (25.49%) and lung (23.53%). Forty-five (88.24%) patients received hydroxychloroquine-based therapy. In addition, 25 of 51 patients died (49%): 12 of 51 (23.53%) owing to cancer and 13 of 51 (25.49%) owing to COVID-19.

**Conclusion:**

The risks for death were related to later onset of treatment for COVID-19, severe/critical COVID-19, age, elevated basal CRP and elevated lactate dehydrogenase.

## Lay abstract

The effect of COVID-19 on cancer patients is of high concern during the COVID-19 outbreak. Here we expand on our initial analysis of 25 patients, presenting 51 patients with COVID-19 and cancer and examining their risk factors for mortality considering indicators such as age and treatment history. We confirm that risk of mortality is greater for cancer patients, and also confirm well-known risk factors for mortality in this group.

A novel coronavirus named coronavirus disease 2019 (COVID-19) emerged in Wuhan, China, in December 2019 and quickly spread globally [1].

Cancer patients are at high risk of acquiring this virus owing to poor general conditions, systemic immunosuppressive state caused by the cancer itself and/or anticancer treatments such as chemotherapy, radiation, surgery, steroids, and so on. In addition, cancer patients have frequent scheduled visits to the hospital and clinics, which can increase the risk of catching COVID-19 [2].

As reported from China, patients with cancer have a markedly elevated risk of intubation, intensive care unit admission or death, both for cancer patients receiving active anticancer treatment and cancer survivors [3]. In oncological units in Italy, clinical consultations for patients not requiring active treatment for cancer and follow-up are performed at wider intervals of time [4–6]. According to Italian oncologists, other types of preventive measures have been established to reduce virus spread; patients are subjected to a triage before admission to the hospital, and more specifically, the triage screening tools used include phone calls, virtual consultations and telemedicine [7,8].

However, the district of Piacenza in the Emilia Romagna Region is very near to the epicenter of the outbreak of COVID-19 in Italy (10 min by car); the catastrophic nature of that North Italy outbreak has been widely publicized [9,10]. Many oncology patients coming from the epicenter of the outbreak of COVID-19 were actively receiving treatment at the oncologic department of the hospital of Piacenza. We recently reported the first 25 cancer patients complicated with COVID-19 [11], and the aims of the present study are to collect the medical information of an additional 51 cancer patients with COVID-19 admitted to the hospitals within the district of Piacenza and to report the clinical characteristics and outcome of these patients. Thus, the total number of cancer patients with COVID-19 hospitalized between 21 February and 4 May 2020 in the district of Piacenza is 76 cases. We report clinical and laboratory data such as CRP and LDH values that could have prognostic significance [12,13]. After all, data on cancer patients and COVID-19 in western countries are still fragmentary and sometimes contradictory [11,14–16]; thus, we believe that more information is likely to be helpful in improving the management of these patients.

## Material & methods

### Patients

We conducted this retrospective study at the three hospitals of the district of Piacenza, Emilia Romagna Region, North Italy. We reviewed the medical records of 973 patients with COVID-19 hospitalized between 4 April 2020 and 4 May 2020; we retrieved a total of 51 cancer cases (5.24%). In all of these patients the COVID-19 infection was diagnosed with laboratory-confirmed SARS-CoV-2 infection, with reverse-transcription PCR in nasopharyngeal swabs. The medical records of patients were analyzed by a team of oncologists, and data were obtained with data collection forms from electronic medical records and included demographic and clinical features, laboratory findings and chest radiological images. The COVID-19 severity was divided into two groups: mild/moderate and severe/critical in accordance with the diagnosis and treatment of COVID-19 guidelines of China [17]. The severe/critical group included patients with respiratory illness defined as an oxygen saturation of 94% or less while they were breathing ambient air, or a ratio of the partial pressure of oxygen to the fraction of inspired oxygen of less than 300 mmHg that required oxygen treatment [18].

### Statistical analysis

Continuous variables were presented by mean values ± standard deviation, and categorical ones were presented by percentages. Categorical variables were analyzed using the chi-square test or Fisher’s exact test, and continuous variables were analyzed using Student’s t-test or the Mann–Whitney test. Variables were included in a univariate Cox analysis using R language with packages ‘survival’ and ‘survminer’. For all Cox regression models, we tested for evidence against the proportional hazard assumption on the basis of Schoenfeld residuals after fitting a model with Cox regression. The results of this regression analyses were reported as hazard ratio (HR) with 95% CI. Time to mortality was analyzed using Kaplan–Meier survival analysis, and the log-rank test was used for comparison between the two groups. A p < 0.05 was considered statistically significant. The Rstudio-1.2.1335 program was used for the analysis.

### Laboratory tests

Nasopharyngeal swab specimens were collected and analyzed with the PCR result according to CDC guidelines [19].

Diagnostic kits for IgM antibodies to *Mycoplasma pneumoniae*, *Chlamydia pneumonia*e and *Legionella pneumophila* was used for detecting three kinds of common respiratory pathogens. Other laboratory tests were part of the standard performed during hospitalization (lymphocytes, CRP, LDH, blood count with formula and platelets, liver, kidney analysis). ECG was performed in all patients, and echocardiogram was performed when required. All patients received radiological evaluation of the chest by x-ray or computed tomography.

### Treatment

Hydroxychloroquine with or without antiviral treatment has been incorporated in our regional guideline to treat COVID-19 [11,20].

During the hospitalization, patients were treated with supportive therapy, antibiotics, oxygen, steroids, heparin and therapy for COVID-19 (lopinavir and ritonavir tablets (800/200 mg daily) plus hydroxychloroquine tablets (400 mg daily) for 7 days or darunavir and cobicistat (800/150 mg daily) plus hydroxychloroquine tablets (400 mg daily for 7 days), or hydroxychloroquine alone (400 mg daily for 7 days).

Three physicians (L Cavanna, I Toscani, M Ambroggi) independently reviewed the data. The study was approved by the Local Ethics Committee of Area Vasta Emila Romagna (institutional review board approval number 494/2020/OSS*/AUSLPC).

## Results

We retrospectively enrolled 51 cancer patients from the 973 COVID-19 cases (5.24%) admitted to three hospitals in the district of Piacenza (Emilia Romagna Region; 290,000 inhabitants), between 4 April 2020 and 4 May 2020. All of these patients were Italian. Clinical and demographic characteristics are summarized in Table 1. The median age of the 51 cancer patients who were COVID-19 positive was 71.02 years (range: 50–86). Thirty-six (70.59%) were male, and 15 (29.41%) were female. The distribution of cancer in 51 patients were gastrointestinal (25.49%), genitourinary (25.49%), lung (23.53%), hematologic (9.8%), breast (7.84%) and cancer of unknown origin (7.84%). The majority of these patients were in advanced stages (68.63% stage IV); however, 21.57% showed no evidence of neoplastic disease, and 24 patients (47.06%) were receiving anticancer active treatment within 1 month of COVID-19. Fourteen patients (27.45%) were treated with chemotherapy, 6 (11.76%) with immunotherapy and 4 (16.67%) with tyrosine kinase inhibitors, whereas 27 patients (52.94%) were off therapy. The clinical symptom of these patients were fever 38°C or higher (100%), dyspnea (70.59%), dry cough (33.33%), fatigue (33.33%) and gastrointestinal symptoms (diarrhea, abdominal pain, nausea; 35.29%). Chest radiological evaluation on admission showed bilateral pneumonia in 86.27% and unilateral pneumonia in 13.73% of these patients. There were 38 (74.51%) patients with comorbidities, and the most common comorbidity was hypertension (66.67%), followed by diabetes (23.53%) and chronic obstructive pulmonary disease (21.57%). Eleven patients (21.60%) showed mild/moderate types of COVID-19, whereas 40 cases (78,40%) showed a severe/critical type. Among the 40 patients with the severe/critical type of COVID-19, 31 (77.5%) had comorbidities, and one or more comorbidities were registered in 84% patients who died. Laboratory findings showed that CRP and LDH values were statistically higher in patients who died (Table 2), whereas there was not a significative difference of mean level of lymphocyte count between patients who died and patients who recovered. Forty-five patients (88.24%) received treatment for COVID-19 (Table 3); 29 patients (56.86%) received darunavir plus cobicistat and hydroxychloroquine, 10 (19.60%) received lopinavir plus ritonavir and hydroxychloroquine, 6 (11.37%) received hydroxychloroquine alone and 6 (11.37%) did not receive anti-COVID-19 therapy because 5 of them worsened quickly after hospital admission and died within 3 days of admission and 1 patient with mild/moderate symptoms of COVID-19 received only supportive therapy. Oxygen therapy was performed in 46 patients (90.2%). The majority of patients (60%) with severe/critical COVID-19 died, whereas 10 of 11 patients (90.91%) with mild/moderate symptoms of COVID-19 recovered. Twenty-five of 51 patients died (49.02%). The cause of death was cancer in 12 of 51 patients (25.51%): 1 patient with mild/moderate and 11 with severe/critical symptoms of COVID-19, whereas for 13 of 51 patients (25.49%) who died, the cause of death was COVID-19 (Table 4). In the noncancer COVID-19 patients, the mortality rate was 20.35%.

**Table 1.** Demographic, clinical and radiological characteristics of hospitalized patients with COVID-19 with different outcomes.

Variable	Cancer patients with COVID-19 (n = 51)	Alive patients (n = 26; 50.98%)	Mortality (n = 25; 49.02%)	p-value
Age (years), mean (range)	71.02 ± 9.36 (50–86)	68.23 ± 9.83 (50–86)	73.92 ± 8.04 (59–84)	**0.03**
– ≤75, n (%)	31 (60.78)	18 (69.23)	13 (52)	0.33
– ≥76, n (%)	20 (39.22)	8 (30.77)	12 (48)	
Sex				
– Male, n (%)	36 (70.59)	16 (61.54)	20 (80)	0.26
– Female, n (%)	15 (29.41)	10 (38.46)	5 (20)	
Tumor site				
– Breast, n (%)	4 (7.84)	2 (7.69)	2 (8)	0.85
– Gastroenteric, n (%)	13 (25.49)	8 (30.77)	5 (20)	
– Genitourinary, n (%)	13 (25.49)	7 (26.92)	6 (24)	
– Hematologic, n (%)	5 (9.80)	3 (11.54)	2 (8)	
– Lung, n (%)	12 (23.53)	5 (19.23)	7 (28)	
– Undefined, n (%)	4 (7.84)	1 (3.85)	3 (12)	
Stage				
– III, n (%)	5 (9.80)	4 (15.39)	1 (4)	0.35
– IV, n (%)	35 (68.63)	16 (61.54)	19 (76)	
– NED, n (%)	11 (21.57)	6 (23.07)	5 (20)	
Cancer therapy				
– Yes, n (%)	24 (47.06)	14 (53.85)	10 (40)	0.48
– No, n (%)	27 (52.94)	12 (46.15)	15 (60)	
Anticancer therapy within 1 month				
– Immunotherapy, n (%)	6 (25)	5 (35.71)	1 (10)	0.24
– Chemotherapy, n (%)	14 (58.33)	6 (42.86)	8 (80)	
– TKI, n (%)	4 (16.67)	3 (21.43)	1 (10)	
Signs & symptoms				
– Fever ≥ 38°C, n (%)	51 (100)	26 (100)	25 (100)	1
– Cough, n (%)	17 (33.33)	10 (38.46)	7 (28)	0.62
– Dyspnea, n (%)	36 (70.59)	16 (61.54)	20 (80)	0.25
– Fatigue, n (%)	17 (33.33)	7 (26.92)	10 (40)	0.98
– Gastrointestinal, n (%)	18 (35.29)	11 (42.31)	7 (28)	0.44
Radiological examination				
– Chest x-ray, n (%)	7 (13.73)	5 (19.23)	2 (8)	0.10
– Chest CT scan, n (%)	44 (86.27)	21 (80.77)	23 (92)	
Interstitial pneumonia				
– Bilateral, n (%)	44 (86.27)	20 (76.92)	24 (96)	0.42
– Unilateral, n (%)	7 (13.73)	6 (23.08)	1 (4)	
Comorbidity				
– Yes, n (%)	38 (74.51)	17 (65.38)	21 (84)	0.27
– No, n (%)	13 (25.49)	9 (34.62)	4 (16)	
COPD, n (%)	11 (21.57)	4 (15.39)	7 (28)	0.45
Diabetes, n (%)	12 (23.53)	4 (15.39)	8 (32)	0.29
Hypertension, n (%)	34 (66.67)	15 (57.69)	19 (76)	0.28
Smoking				
– Yes, n (%)	20 (39.22)	8 (30.77)	12 (48)	0.33

COPD: Chronic obstructive pulmonary disease; COVID-19: Coronavirus disease 2019; NED: No evidence of disease; TKI: Tyrosine kinase inhibitor.

Significant p-values reported in bold terms.

**Table 2.** Laboratory findings of cancer patients with COVID-19.

Variable	Cancer patients with COVID-19 (n = 51)	Alive patients (n = 26, 50.98%)	Mortality (n = 25, 49.02%)	p-value
Lymphocytes, mean (range)	1.12 ± 2.26 (0.05–16.5)	0.74 ± 0.28 (0.2–1.2)	1.52 ± 3.21 (0.05–16.5)	
≥0.8 × 10^3^/ϻL, n (%)	22 (43.14)	12 (46.15)	10 (40)	0.76
<0.8 × 10^3^/ϻL, n (%)	29 (56.86)	14 (53.85)	15 (60)	
Neutrophil count, mean (range)	6.48 ± 5.62 (0.4–27.67)	6.31 ± 5.99 (1.32–27.67)	6.64 ± 5.32 (0.4–21.47)	
≤2 × 10^3^/ϻL, n (%)	8 (15.69)	4 (15.38)	4 (16)	0.58
2–8 × 10^3^/ϻL, n (%)	29 (56.86)	16 (61.54)	13 (52)	
≥8 × 10^3^/ϻL, n (%)	14 (27.45)	6 (23.08)	8 (32)	
Platelet count, mean (range)	214 ± 125 (30–558)	228 ± 140 (41–558)	201.08 ± 109.70 (30–413)	
<150 × 10^3^/ϻL, n (%)	19 (37.25)	10 (38.46)	9 (36)	0.69
≥150 × 10^3^/ϻL, n (%)	32 (62.75)	16 (61.54)	16 (64)	
CRP, mean (range)	10.38 ± 6.29 (0.97–29)	8.51 ± 5.64 (0.97–19)	12.32 ± 6.45 (2.7–29)	
≤10 mg/dL, n (%)	27 (52.94)	17 (65.39)	10 (40)	**0.02**
≥11 mg/dL, n (%)	24 (47.06)	9 (34.61)	15 (60)	
LDH, mean (range)	475 ± 405 (145–2781)	311.62 ± 128.12 (145–593)	646 ± 516.22 (160–2781)	
<250 U/L, n (%)	12 (23.53)	10 (38.46)	2 (8)	**0.004**
≥250 to <450 U/L, n (%)	17 (33.33)	12 (46.15)	5 (20)	
≥450 U/L, n (%)	22 (43.14)	4 (15.38)	18 (72)	

COVID-19: Coronavirus disease 2019.

Significant p-values reported in bold terms.

**Table 3.** Treatment of cancer patients with COVID-19.

Therapy	Cancer patients with COVID-19 (n = 51)	Alive patients (n = 26, 50.98%)	Mortality^‡^ (n = 25, 49.02%)	p-value
Anti-COVID-19 treatment				
– Darunavir + cobicistat (800/150 mg daily) + hydroxychloroquine (400 mg daily), n (%) for 7 days	29 (56.86)	17 (65.39)	12 (48)	0.21
– Lopinavir + ritonavir (800/200 mg daily) + hydroxychloroquine (400 mg daily), n (%) for 7 days	10 (19.60)	6 (23.08)	4 (16)	
– Hydroxychloroquine (400 mg daily), n (%) for 7 days	6 (11.77)	2 (7.69)	4 (16)	
– No treatment, n (%)^†^	6 (11.77)	1 (3.84)	5 (20)	
Time from symptoms to treatment (days), mean (range)	5.35 ± 2.83 (1–11)	3.04 ± 1.25 (1–7)	7.76 ± 1.81 (5–11)	**<0.001**

† Five patients died within 3 days of admission, and 1 had mild/moderate symptoms of COVID-19.

‡ For 12 of 25 dead patients (48%), the cause of death was cancer progression.

COVID-19: Coronavirus disease 2019.

Significant p-values reported in bold terms.

**Table 4.** Outcome of COVID-19 cancer patients based on severity of type of COVID-19.

Outcome	Mild/moderate type (n = 11, 21.57%)	Severe/critical type (n = 40, 78.43%)	p-value
Alive, n (%)	10 (90.91)	16 (40)	**0.005**
Dead, n (%)	1 (9.09)	24 (60)	
Cause of death			
– Cancer progression, n (%)	1 (100)	11 (45.83)	
– COVID-19, n (%)	0 (0)	13 (54.16)	

Note: Significant p-values reported in bold terms.

**Figure 1.**
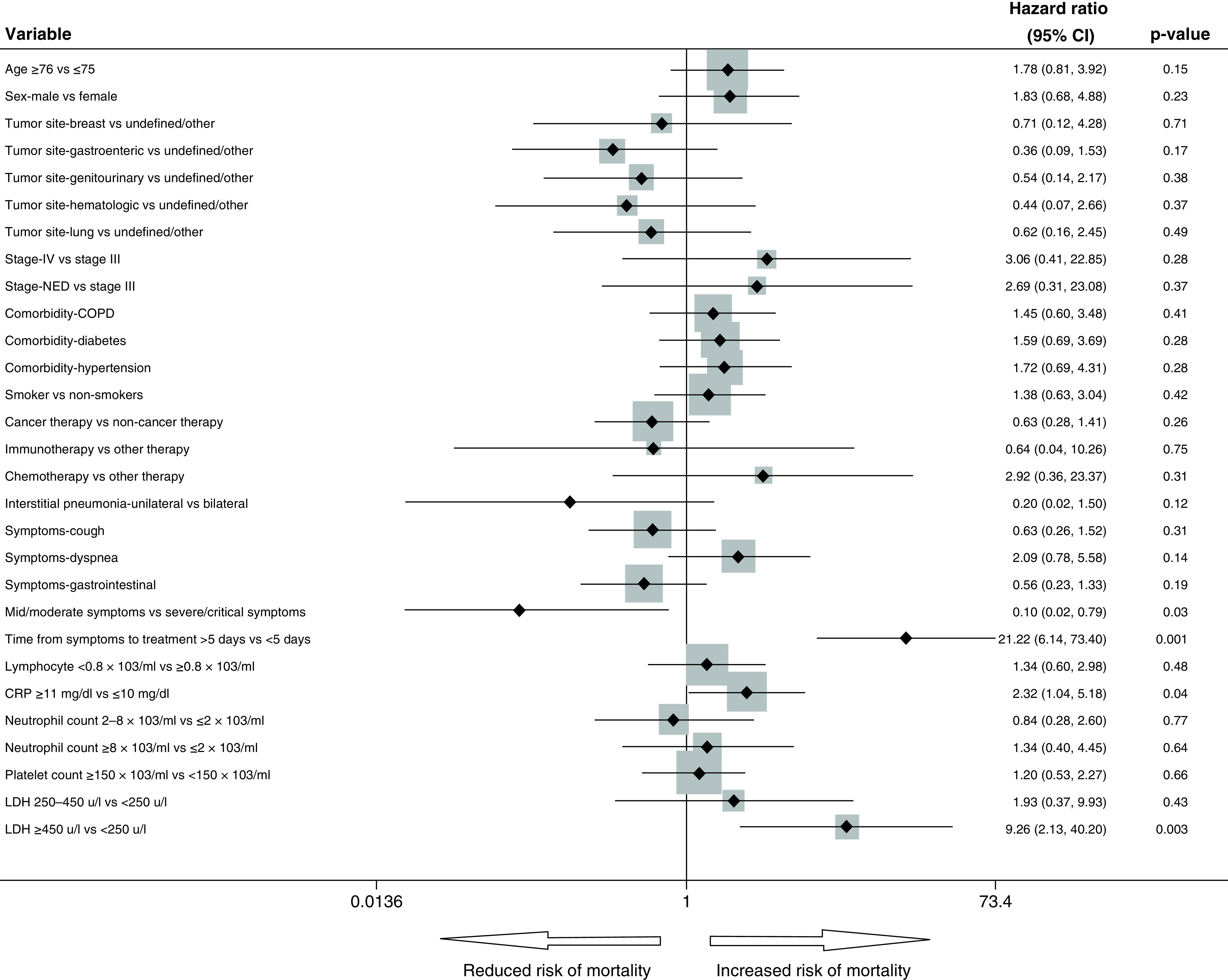
Cox regression analysis of potential prognostic factors. COPD: Chronic obstructive pulmonary disease; NED: No evidence of disease.

**Figure 2.**
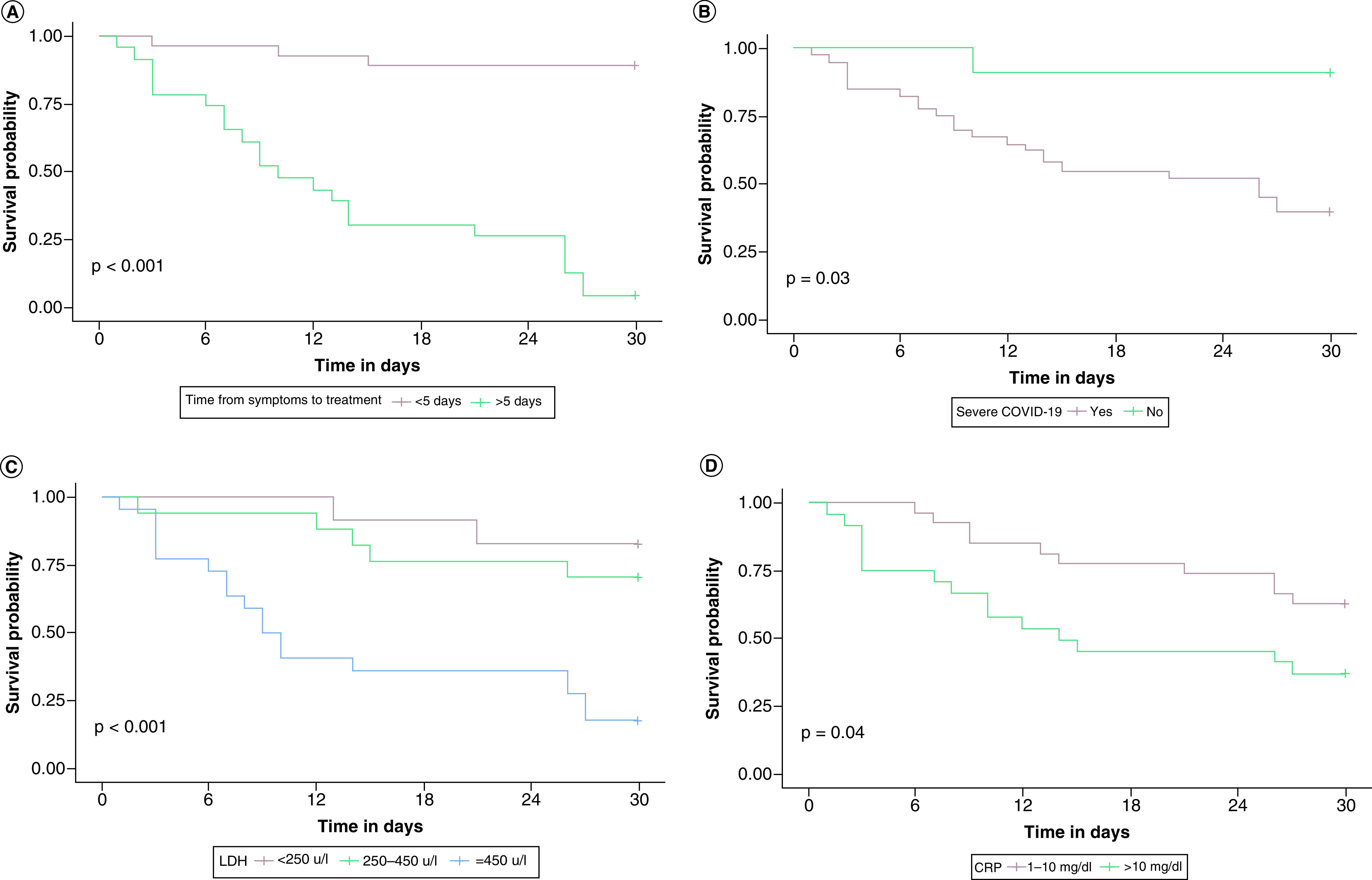
Kaplan–Meier survival plots for different prognostic factors. The figure displays the Kaplan–Meier survival plots according to time of symptoms to treatment **(A)**, COVID-19 severity **(B)**, LDH **(C)** and CRP **(D)**. COVID-19: Coronavirus disease 2019.

In univariable analysis, hazard of death was higher in patients with high basal CRP values (>11 mg/dL) (HR: 2.32; 95% CI: 1.04–5.18; p = 0.04), high basal LDH values (>450 U/L) (HR: 9.26; 95% CI: 2.13–40.20; p = 0.003), patients who started treatment more than 5 days after the onset of symptoms (HR: 21.22; 95% CI: 6.14–73.4; p < 0.001) and lower in patient with mild/moderate type of COVID-19 compared with patient with severe/critical type (HR: 0.10; 95% CI: 0.02–0.79; Figure 1). Kaplan–Meier survival plots for these prognostic factors are shown in Figure 2. Multivariate analysis was not performed to make sure that these were independent prognostic factors.

## Discussion

The clinical characteristics of 51 cancer patients with laboratory-confirmed COVID-19 from three hospitals in the district of Piacenza (Italy) are described.

The complex care of cancer patients requiring multidisciplinary approaches with surgery, chemotherapy, targeted therapy, immunotherapy and radiation during the COVID-19 pandemic poses unprecedent ed challenges. Oncologists, in addition to caring for cancer patients, must also protect patients and their caregivers from unnecessary exposure [21]. It has been reported from China that approximately 1% of patients infected with COVID-19 had cancer, and it was five-times higher than the general incidence in China [22]. In addition, a report from Italy provides evidence that approximately 20% of deceased patients with COVID-19 had a cancer in the past 5 years [15]. We recently reported a small series of 25 cancer patients and COVID-19 [11]. All of these 25 patients showed fever, cough and shortness of breath. Nine of them (36%) died, whereas 16 (64%) overcame the infection; prognosis was related to older age, gender and COVID-19 treatment. In a recent report from Wuhan [23], the infection rate of COVID-19 in cancer patients was higher (3.3%) than the general population. In addition, in this study, 36.5% of cancer patients with COVID-19 were severe/critical cases; this percentage is higher than in the general population, and it is well known that the mortality rate is higher in patients with severe/critical COVID-19. However, the mortality rate of patients with COVID-19 and cancer versus patients without cancer is still controversial, as reported previously [24]. In our study, the medical records of 973 patients with COVID-19 admitted to the hospitals of Piacenza were reviewed and 51 cases with cancer were retrieved (5.24%). This infection rate of COVID-19 in our series of cancer patients is much higher than the general population, as reported previously [22,23,25–27]. It must be emphasized that Italy was the first country in Europe to experience an outbreak of COVID-19, and the city of Piacenza is very near to the epicenter of the Lombardy outbreak (only 10 min by car). In our series, the median age of patients with cancer and COVID-19 was higher when compared with other reported series: 71.02 years (range: 50–86) versus 63 years (range: 56–70) [26] or 65 years (range: 34–98) [23]. It is well known that cancer patients may be susceptible to infection during the COVID-19 pandemic secondary to their impaired immune function [23].

However, it must be emphasized that it has been reported that cancers such as colon, breast and lung cancer do not typically cause immune suppression that is not treatment related [27]. The main symptoms of our patients were fever 38°C or higher (100%), dyspnea (70.59%) cough and fatigue (33.33%) that were similar to those of previous reports and of the general population infected by COVID-19 [26]. Among the 51 cancer patients with COVID-19, the main sites of cancer were gastrointestinal (25.49%) and genitourinary (25.49%), followed by lung (23.53%), then breast and undefined origin (7.8%) and hematological (9.8%) cancers. Thirty-eight of 51 patients (74.51%) showed comorbidities, with hypertension being the most common in 34 of 51 patients (66.67%), followed by diabetes in 12 of 51 patients (23.53%) and chronic obstructive pulmonary disease in 11 of 51 cases (21.57%); those data are similar to the previous report [23].

Comorbidities seem to play an important role in the prognosis of cancer patients with COVID; in fact, 84% of our patients who died had one or more comorbidities as reported previously [26]. The majority of patients were male. Thus, that could have contributed to the high death rate [28].

In a small series of 28 cancer patients with COVID-19, it was identified that recent anticancer treatment within 14 days of infection, such as chemotherapy, immunotherapy or radiation, was an independent predictor of death or other severe events with a HR greater than 4 [26].

However, in the largest multicenter studies, no interactions between anticancer treatment within 4 weeks prior to COVID-19 diagnosis and COVID-19-related mortality were found [29,30]. Our data are in agreement with these studies, since in our series 24 of 51 (47.06%) cancer patients received anticancer treatment within 4 weeks before testing positive for COVID-19, and no interaction was found in patients’ outcome. However, more recent results presented at European Society for Medical Oncology (ESMO) by CCC19 have suggested that chemoimmunotherapy was associated with a high mortality rate [31].

Currently, there are no clinical data on the efficacy and safety of antiviral prophylaxis for COVID-19 in cancer patients and little is known about the treatment of COVID-19 in cancer patients [32].

Although no vaccine or specific antiviral treatment for COVID-19 has yet been demonstrated to be effective in Phase 3 randomized clinical trials, hydroxychloroquine with or without antiviral treatment has been incorporated in some national or regional guidelines to treat COVID-19 [11,20], and our patients with cancer were treated for COVID-19 as were noncancer COVID-19 patients, with hydroxychloroquine-based therapy [11,20]. Hydroxychloroquine has received worldwide attention as a potential treatment for COVID-19 because of positive results from some studies [33,34]. However, other studies do not support its use in patients admitted to the hospital with COVID-19 [35,36]. Moreover, recent positive research supports the use of dexamethasone in COVID-19 patients [37].

## Conclusion & future perspective

In this retrospective study, we confirmed the well-known risk factors of death for cancer patients and COVID-19, such as advanced age, severe/critical type of COVID-19 and laboratory data (elevated CRP, LDH). In addition, we acknowledge some limitations to the study (retrospective study, small size, monoinstitutional study and the disparity between the type of cancers that our study included [most common were gastrointestinal, genitourinary and lung] versus the normal incidence of cancers) may indicate that this study may be not representative of the effects of COVID-19 on cancer. With the limitations of univariate analysis, we reported that treatment with hydroxychloroquine-based therapy was significantly associated with favorable outcome.

The COVID-19 outbreak is a major global public health pandemic, and it can have catastrophic consequences for cancer patients. We believe that physicians, caregivers and patients, while waiting for a vaccine, should research prevention, early diagnosis and therapeutic pragmatic approaches to improve cancer patient outcome. Oncologists, in addition to providing care for cancer patients, must also protect patients and their caregivers from unnecessary exposure, particularly considering that mortality from COVID-19 is greater in cancer patients than for patients without cancer.

**Summary points**

- Mortality for coronavirus disease 2019 (COVID-19) appears greater in cancer patients when compared with noncancer patients.
- Cancer patients receiving anticancer treatment in the previous 4 weeks were not at an increased risk of mortality from COVID-19 when compared with patients not on active treatment.
- High basal C-reactive protein and lactate dehydrogenase values showed an unfavorable prognostic role.
- Mortality was driven by age (specifically older age) and severity of COVID-19: 90.91% of patients with mild/moderate type overcame COVID-19, whereas 60% with severe/critical COVID-19 died.
- With the limitations of univariate analysis, the treatment of COVID-19 within 5 days of symptoms with a hydroxychloroquine-based therapy improved the outcome of infected cancer patients.
